# Transition to Parenthood and Marital Satisfaction: A Meta-Analysis

**DOI:** 10.3389/fpsyg.2022.901362

**Published:** 2022-07-20

**Authors:** Ionela Bogdan, Maria Nicoleta Turliuc, Octav Sorin Candel

**Affiliations:** Department of Psychology, Alexandru Ioan Cuza University of Iaşi, Iaşi, Romania

**Keywords:** marital satisfaction, transition to parenthood, meta-analysis, cross-partners associations, moderators

## Abstract

The transition to parenthood is a major life event characterized by profound changes for a considerable number of people. Previous meta-analyses summarized the results obtained by various researchers in the first year and, respectively, in the first 2 years postpartum, globally. The current study adds to the literature by testing the changes from 12 to 24 months, the cross-partner associations and the analysis of different moderators. The aims of this present meta-analysis are to investigate the decrease in marital satisfaction during the first and second year postpartum, to examine cross-partner associations of the decline in postpartum marital satisfaction, and to investigate the potential moderating variables of this decrease. Forty-nine studies (97 samples of parents and 9 samples of non-parents) that fit our criteria are included in the meta-analysis. The data analysis was performed using meta-analytic techniques. Marital satisfaction has a medium decrease between pregnancy and 12 months postpartum, and a small decline between 12 and 24 months postpartum for both genders. In a similar period with first year postpartum, non-parents present a small decline in marital satisfaction. Moreover, the analysis of the dyadic studies data shows cross-partner associations, confirming that one partner’s satisfaction has a steeper decline when the other partner’s satisfaction presents a steep decrease. The decrease in marital satisfaction does not stop after the first postpartum year, and the coss-partners associations are present. Theoretical and therapeutic implications are also discussed.

## Introduction

Married people are generally happier than unmarried or cohabiting people (Vanassche et al., 2013). Although the protective role of marriage has been well-established over time (Waite and Lehrer, 2003; Rendall et al., 2011), it has also been proven that those who have an unhappy marriage suffer from more medical conditions, and their life expectancy is lower than that of unmarried, divorced or widowed individuals (Lawrence et al., 2019). Moreover, marital satisfaction is not stable over time, White and Edwards (1990) describe a “U”-shaped marital satisfaction trajectory. The research of the early decline of marital satisfaction presents different results. The two existing meta-analyses on this topic have indicated small (Mitnick et al., 2009) vs. moderate (Twenge et al., 2003) decreasesin marital satisfaction after the first child’s birth. Moreover, in one meta-analysis, similar trajectories were found for parents and non-parents (Mitnick et al., 2009), while in the other, less marital satisfaction of parents was found compared to non-parents (Twenge et al., 2003).

Building on these findings, we first aimed to deepen the trajectory of marital satisfaction in men and women during the transition to parenthood. Thus, we investigated the decrease in marital satisfaction in the first and in the second year postpartum using a sample of longitudinal studies with two or three marital satisfaction measurements: during the late pregnancy, at the end of the first year and at the end of the second year postpartum. The previous meta-analyses presented the decrease in marital satisfaction in the first year, respectively, in the first 2 years postpartum globally. This is the first attempt to analyze the trajectory of marital satisfaction in the first year, and in the second year separately, to see if the decrease in the second year is similar or not to than in the first one. Furthermore, we include in our analysis non-parent samples in order to test if the couples without children have the same marital satisfaction trajectory in a similar period of their marriage.

Although empirical studies highlight cross-partner associations, none of the existing meta-analyzes have analyzed this aspect. Therefore, our second aim was to investigate whether one partner’s satisfaction has a steeper decline when the other partner’s satisfaction also has a more pronounced decrease. Moreover, there is little evidence of how marital satisfaction changes during the transition to parenthood depending on the participants’ demographic characteristics. Subsequently, our third aim was to investigate the possible moderators of the satisfaction’s trajectory during this period.

### Transition to Parenthood and Marital Satisfaction

The transition to parenthood is a major life event characterized by profound changes for a considerable number of newlyweds. Even though the arrival of a baby is often a joyful event, it can affect interpersonal resources. The transition to parenthood is a transformative experience that changes the self-concept, social roles and daily routines (Saxbe et al., 2018). Therefore, it is associated with more stress and decreases both partners’ marital satisfaction (Mirowsky and Ross, 2002). The quality of the spouses’ relationship depends on their adaptation to this new status. The process of intense adjustment to parenthood begins when the woman becomes pregnant and ends when the child reaches the age of two (Doss et al., 2009).

A growing number of studies had looked at different characteristics of couples during the early transition to parenthood, drawing some conclusions regarding the trajectory of marital satisfaction. Moreover, the two previous meta-analyses allowed us to test and to build upon their results (Twenge et al., 2003; Mitnick et al., 2009). As mentioned, Mitnick et al. (2009) found a small decrease in marital satisfaction during the first 11 months postpartum. Treating globally the first 2 years after the first child’s birth, Twenge et al. (2003) indicated that marital satisfaction substantially decrease. Their conclusions were also different when comparing parents with non-parents.

The empirical research over the last decades has also reached some different results. While several studies illustrate that transition to parenthood brings a decrease in marital satisfaction (e.g., Doss et al., 2009; Simonelli et al., 2016; Bäckström et al., 2018), other research shows that a decrease in marital satisfaction is common in newlyweds with or without children (Lawrence et al., 2008). Moreover, more recent studies on marital satisfaction of newlyweds highlight that trajectories depend on the initial level of marital satisfaction. Therefore, people who report a low initial level of marital satisfaction suffer from a more significant decrease in the first years of marriage, while people who indicate a medium or high level of marital satisfaction remain at their initial level of their marital satisfaction (Birditt et al., 2012; Williamson and Lavner, 2020). However, the research on newlyweds contains no information on whether the participants became parents during the study. Furthermore, a review that includes 14 empirical studies on newlyweds concludes that a decrease in marital satisfaction is due to a low initial level of marital satisfaction or essential experiences like the transition to parenthood (Proulx et al., 2017).

Based on previous research findings, Lawrence et al. (2008) identified two perpectives of the fundamental nature of marital satisfaction change during the transition to parenthood. The first assumes that the partners experience a qualitative change in their relationship. Their marital satisfaction decrease and the decline is sudden, significant and persistent in time. The second refers to the minor, temporary and quantitative character of the decline in marital satisfaction. The satisfaction decrease depends on the partners’ capacity to manage the transition to the new couple’s stage (Lawrence et al., 2008). The attachment theory (Bowlby, 1988) deepens the explanation of these perspectives. A partner’s secure attachment has positive effects on marital satisfaction during postpartum through the high support he/she gives and the optimistic expectations from the partner. This behavior may explain the second perspective. In contrast, an insecure attachment negatively influences couple satisfaction due to partner’s demand for attachment and the critical manner he/she ask for it (Cowan and Cowan, 2000; Velotti et al., 2011). When both partners have an insecure attachment, the decrease in marital satisfaction is considerably higher than in couples in which both partners have a secure attachment or one of them has a secure attachment (Velotti et al., 2011). Furthermore, in threatening and stressful periods, the attachment system is activated, and anxiety and avoidance increase. This may explain the findings within the first perspective of sudden changes of marital satisfaction.

Thus, building on the previous studies and on their slightly different results, we aim to investigate whether the decrease in marital satisfaction characterizes only the first-time parents, and how large it is. According to our fist aim, based on previous research results and to addressed the gap in the literature we hypothesized that: *(H1) Marital satisfaction suffers a decrease from pregnancy up to 12 months postpartum for both men and women; (H2) The decrease in marital satisfaction continues between 12 and 24 months postpartum for both men and women; (H3) The trajectories of marital satisfaction are different in parents and non-parents. The decrease is higher for parents compared to non-parents.*

### Cross-Partner Associations

The family systems’ approach assumes that families are emotional units of interdependent individuals (Minuchin, 1974). Not surprisingly, previous studies highlighted the need to focus on dyadic data analysis (Gameiro et al., 2011). The dyadic studies’ results underline the partner effects (e.g., Meijer and van den Wittenboer, 2007; Don and Mickelson, 2014), suggesting that some of the husband’s characteristics have an essential role in the wife’s marital satisfaction, and vice-versa (Don and Mickelson, 2014). Also, the emotional state of one of the partners influences the marital satisfaction of the other partner. For example, people characterized by low levels of a negative emotional state in the third trimester of pregnancy are those whose partners report higher marital satisfaction levels (Bower et al., 2013). Moreover, during the transition to parenthood, the partners tend to have a similar marital satisfaction trajectory marital satisfaction (Don and Mickelson, 2014). Furthermore, none of the existing meta-analyses the cross-partner associations. Therefore, the second aim of this meta-analysis is to test the cross-partner associations: whether a partner’s decrease in marital satisfaction has a significant impact on the other partner’s decrease in marital satisfaction 12 months after birth. Thus, we formulated the following hypothesis: *(H4) The steeper decrease in one partner’s satisfaction is associated with the steeper decrease in the other partner’s level of satisfaction.*

### Moderators

Marital satisfaction is influenced by demographic data, like gender (Elek et al., 2003; Twenge et al., 2003), age (Trillingsgaard et al., 2014), or length of relationship (Doss et al., 2009). Therefore, we analyze the extent to which all these variables, described below, moderates the decrease in marital satisfaction separately in the first, and in the second year after the child’ birth.

#### Gender

Research supports the idea that the postpartum marital satisfaction of mothers is more affected than that of fathers (Twenge et al., 2003). Don and Mickelson (2014) showed that almost 80% of first-time mothers suffer a moderate decrease in marital satisfaction, while 51% of fathers cross a moderate decline, and 49% endure a milder decrease. Other research also indicates a more sudden decrease for women and a gradual decline in men (Belsky and Hsieh, 1998; Grote and Clark, 2001) or a notable decrease for mothers and an unchanged marital satisfaction for fathers (Lawrence et al., 2008).

#### Age

It seems that older women have a more challenging time adapting to their new role immediately after childbirth than the younger ones (Lederman et al., 1981). After a few months, they recover this difference and present better adaptation (Robinson et al., 1988). Moreover, at 12 months postpartum, older women are more satisfied with their husband’s domestic activities than younger women (Robinson et al., 1988). Childbirth at a younger age is associated with lower marital satisfaction (Doss et al., 2009; Trillingsgaard et al., 2014).

#### Relationship Length

It has been shown that having a child earlier in a marriage predicts more significant decreases in marital satisfaction (Doss et al., 2009). The birth of the first child in the first 3 years of marriage seems to represent a high risk of couple dissolution (Belsky and Rovine, 1990).

#### Measurement

The instruments used in investigations to measure marital satisfaction are generally standardized scales (e.g., Couples Satisfaction Index, Funk and Rogge, 2007; Marital Adjustment Test, Locke and Wallace, 1959; Quality of Marriage Index, Norton, 1983; Relationship Assessment Scale, Hendrick et al., 1998), but by far the most commonly used is the Dyadic Adjustment Scale (DAS; Spanier, 1976). If some decades ago, DAS was applied in over 1,000 studies (Spanier, 1985), today it can be challenging to precisely determine the number of studies that have used this scale. Nevertheless, due to the high number of studies using DAS, we would like to see if this instrument moderates the decline of marital satisfaction.

According to the third aim of this study we hypothesized that: *(H5) The decline in marital satisfaction is moderated by gender, age, relationship length and the instrument used for measuring marital satisfaction. The decrease is higher for women than men, for the younger parents compared to older parents, and for parents having a shorter length of their marital relationship than those having the child later in their marriage. Also, the reported decrease is lower when using the Dyadic Adjustment Scale (DAS; Spanier, 1976) than on other scales.*

## Method

### Literature Search

The meta-analysis was conducted according to the PRISMA 2020 recommendations (Page et al., 2021). We conducted an electronic search using the following databases: PsycInfo, Proquest, Scopus, Web of Science, DOAJ, and Google Scholar. The keywords used individually or in different combinations were: a. “transition,” “parenthood,” “parental status,” “first childbirth,” or “first child,” and b. “marital satisfaction,” “relationship satisfaction,” “marital quality,” and “relationship quality.” The literature search was conducted in five waves (September 2019, April 2020, September, 2020, April 2021, and December, 2021).

### Inclusion and Exclusion Criteria

The inclusion criteria of the current meta-analysis are: (1) the studies of first-time parents included self-reported measures of prepartum marital satisfaction and a similar measure at least one postpartum time, up to 24 months, (2) the studies contained the necessary statistical data allowing us to compute the effect sizes (means and standard deviation for both measurements); (3) the studies were published in peer-reviewed journals; (4) the studies were published in English; (5) for intervention studies targeting marital satisfaction, we eliminated the studies that did not include a control group. Given that we were interested in the mean difference between two dependent measures (the satisfaction level of the same person, at two different time points), the correlation between the two measures was also needed. However, not all studies reported the correlation. To avoid the exclusion of the studies that did not report the correlation, we computed a mean correlation for the whole sample and used it when necessary. Additionally, we included four studies with at least two-time measurements of marital satisfaction in couples without children.

### Effect Size Calculation

We computed effect sizes (standardized mean gain scores) from the raw mean scores, standard deviations and correlations between measurement at the two time points. Given that not all the studies offered the necessary correlations when needed, we used the average correlation coefficient from all the included studies. First, we computed the overall effect size (Hedge’s g) to show the difference between pregnancy and postpartum marital satisfaction up to 12 months after the first child’s birth. Second, we computed the effect size (Hedge’s g) to show the difference in marital satisfaction between the first and the second year postpartum. We computed the overall size effects for mothers, fathers and couples (samples that did not differentiate between genders).

Finally, we wanted to verify whether one partner’s decrease in satisfaction influences the other partner’s decrease in satisfaction. Only the studies that reported data for both partners were included in this analysis. For this, each couple was regarded as a unit of observation, and several steps were necessary. To test this relationship for the wives, we first calculated the effect size for the husbands’ satisfaction decrease. Thus, we obtained a separate effect size for each sample included in the analysis. Then, we computed a meta-regression where we included the husband’s effect sizes as a supplementary *explanatory variable* predicting the outcome variable and the wives’ decrease in satisfaction. The same method was used when assessing the husbands’ decrease in satisfaction.

### Meta-Analytic Strategy and Analyses

We conducted the meta-analyses using the Meta-Essentials (Suurmond et al., 2017). This free tool consists of a set of workbooks designed for Microsoft Excel that, based on the researcher’s input, automatically produces all the required statistics, tables and figures (van Rhee et al., 2015). In terms of usability, it is a simpler tool for conducting meta-analyses because it does not require any coding or programming knowledge and it can handle various effect sizes and most of the common meta−analysis methods. This tool was used in various highly impact studies over the last years (Kim et al., 2019; Kolte et al., 2019; such as Candel and Turliuc, 2019). In this study, the models were tested for differences among dependent groups with continuous data. We tested random-effects’ models, because they allow for wider generalization (Borenstein et al., 2010). The meta-regression analyses were performed using the free JASP software (van Doorn et al., 2021). For the heterogeneity analysis (how consistent the results were across the analysis), we used the tau-squared and the Q-tests (which, when significant, indicate heterogeneity in the sample) and I^2^ percent, which is a better-suited test for larger samples (Higgins et al., 2003). I^2^ takes values from 9 to 100%, with 25, 50, and 75%, meaning low, medium and high heterogeneity. For bias detection, we used the Egger t-test, which, when significant (*p* < 0.05), indicates bias in the analysis (Egger et al., 1997). To further analyze the publication bias, we used the trim-and-fill method as well as the Orwin Fail-Safe N (Orwin, 1983; Duval and Tweedie, 2000).

### Transparency and Openness

We describe the way we obtained our sample size, the literature search including all inclusion and exclusion criteria. We calculated effect sizes using the Meta-Essentials (Suurmond et al., 2017) and JASP software (van Doorn et al., 2021) for meta-regressions and followed Cohen’s (1988) recommendation for the significance of effect sizes. The datasets are publicly available (Open Science Framework) and can be consulted by accessing the following link.^1^ This investigation was not pre-registered.

## Results

**FIGURE 1:**
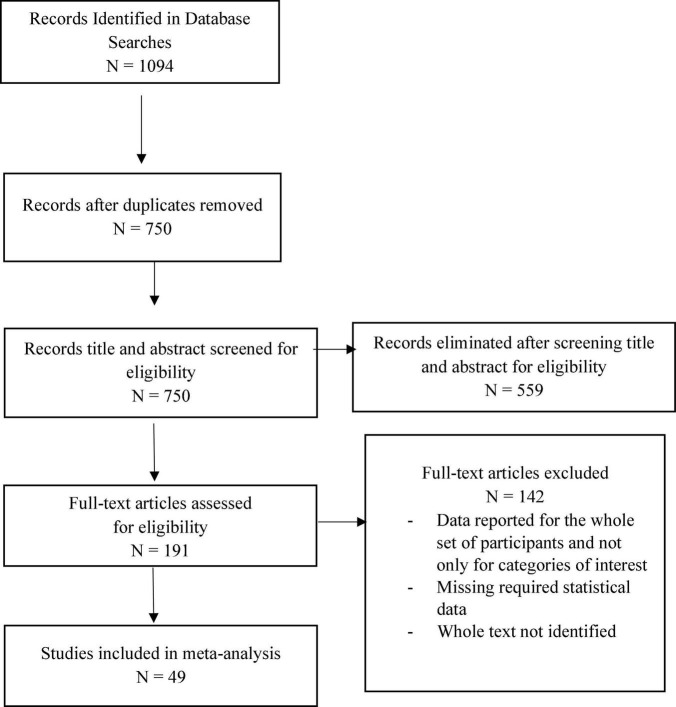
PRISMA flow diagram.

We evaluated the identified samples based on the inclusion criteria and excluded samples that failed to meet the inclusion criteria. A flowchart depicting this process is shown in Figure 1. The list of studies included in this meta-analysis can be consulted in Supplementary Material.

The final sample consisted of 49 studies (45 studies were reporting data for parents only, 1 study for non-parents only and 3 studies for both parents and non-parents), with 106 independent samples (97 samples of parents and 9 samples of non-parents), with a total of 145,139 participants. From these, 4,790 were men and 139,956 were women. The mean age for the whole sample was 29.11 years (*SD* = 2.39). The mean age for men was 29.96 years (*SD* = 1.74) and for women 28.12 (*SD* = 2.21). The mean relationship length was 52.68 months (*SD* = 17.36) for the entire sample, 51.15 months (*SD* = 16.95) for men, and 53.96 months (*SD* = 18.41) for women. In the parent group, 71 samples used dyadic data. Most of the studies were conducted in North America (19 studies) and Europe (14 studies).

### Marital Satisfaction in Parents’ and Non-parents’ Samples (T0–T1)

Firstly, we calculated whether the parents’ and non-parents’ satisfaction decreased between the prepartum assessment and the postpartum assessment, up to 12 months postpartum (or a similar time frame for non-parents) (see Table 1 for the results).

**TABLE 1:** Effect size for the satisfaction decrease in the parents’ and non-parents’ samples.

	Estimates							
Measures	K	N	g (SE)	95% CI LL	95% CI UL	τ ^2^	Q	I^2^ (%)
**First year**								
*Parent sample*								
Women satisfaction change	50	139,791	–0.31*** (0.03)	–0.38	–0.25	0.01	528.16***	90.91
Men satisfaction change	45	4,625	–0.29*** (0.03)	–0.36	–0.22	0.04	238.65***	81.56
Couple satisfaction change	3	291	–0.28* (0.08)	–0.62	–0.06	0.01	4.73	57.71
*Non-parent sample*^a^**								
Women satisfaction change	4	165	–0.12* (0.04)	–0.24	–0.01	0.00	.73	0
Men satisfaction change	4	165	–0.13* (0.04)	–0.24	–0.02	0.00	.71	0
**Second year**								
*Parent sample*								
Women satisfaction change	15	136,337	–0.16*** (0.02)	–0.24	–0.12	0.00	158.47***	91.17
Men satisfaction change	11	1,445	–0.14* (0.06)	–0.27	–0.02	0.03	46.19***	78.35
**Fist 2 years**								
Total satisfaction change	26	137,779	–0.37** (0.04)	–0.43	–0.32	0.01	367.29***	93.19

K, number of effect sizes; N, sample size; g, Hedge’s g; CI, confidence interval; Q, ratio of variation to within-study error; τ^2^ and I^2^, between-study variance. *p < 0.05; **p < 0.01; ***p < 0.001.

*^a^One study did not differentiate between mothers and fathers and was not included in the analysis.*

We used Cohen’s (1988) recommendation regarding the magnitude of effect sizes, namely 0.2 for a small effect, 0.5 for a medium effect and 0.8 for a large effect. The first hypothesis was supported. Overall, the marital satisfaction of the participants from the parents’ sample decreased significantly from T0 to T1 (for mothers: Hedge’s g = –0.31, *p* < 0.001; for fathers: Hedge’s g = –0.29, *p* < 0.001; for couples: Hedge’s g = –0.28, *p* < 0.05). All the effect sizes were medium. The results for mothers and fathers also showed significant heterogeneity in the distribution of effect sizes across the included samples (for mothers: *Q* = 528.16, *p* < 0.001, *I*^2^ = 90.91%; for fathers: *Q* = 238.65, *p* < 0.001, *I*^2^ = 81.56%). The participants from the non-parents’ sample also showed a significant decrease in marital satisfaction, but the effects sizes for this decrease were small (for mothers: Hedge’s g = –0.12, *p* < 0.05; for fathers: Hedge’s g = –0.13, *p* < 0.05).

Based on the significant heterogeneity of the sample, we computed a series of moderation analyses for the parents’ sample.

#### Gender

For the first of these analyses, we used sub-group analysis to test gender as a potential moderator. The results for mothers and fathers are displayed in Table 1. By using heterogeneity Q-test significance, determined by the degree to which the confidence intervals for two or more moderator subgroups overlapped, we observed that the difference between men and women was not significant (*Q* = 0.20, *p* = 0.65).

#### Measurement

We used the type of instrument used for measuring marital satisfaction as a potential moderator for the decrease in marital satisfaction (see Table 2). We observed that there are no significant differences (*Q* = 0.50, *p* = 0.47) in the decrease in marital satisfaction from T0 to T1 between the cases when the DAS scale was used and to the cases where marital satisfaction was measured with another instrument.

**TABLE 2:** Sub-group analyses to test for moderation in the decrease in marital satisfaction (categorical moderators).

	Subgroup summary information				Q-test for heterogeneity		
Moderator	g	95% CI	P	K	Q	P	K
**First year**							
*Satisfaction’s measure*							
DAS	–0.24	[–0.44, 0.05]	0.000	38	1.38	0.24	97
Other	–0.27	[–0.48, –0.07]	0.000	59			
**Second year**							
*Satisfaction’s measure*							
DAS	–0.11	[–0.15, –0.03]	0.000	11	6.50	0.01	26
Other	–0.19	[–0.26, –0.12]	0.000	15			

*g, Hedge’s g; CI, confidence interval; P, level of statistical significance for the aggregated effect size or heterogeneity Q-test; K, number of samples in the moderator subgroup; Q, the Q-value for the heterogeneity Q-test for between-subgroup differences with K – 1 degrees of freedom.*

#### Age

To test the moderating effect of the participants’ age, we conducted a meta-regression where we introduced age as an independent variable (see Table 3). The results show a non-significant effect of age on the decrease in marital satisfaction (*b* = –0.002, S.E. = 0.02, *p* = 0.91).

**TABLE 3:** Meta-regression to test for moderation in the decrease in marital satisfaction (continuous moderators).

Moderator	K	Estimate	S.E.	z	p
**First year**					
Age	89	–0.002	0.02	–0.10	0.91
Relationship length	57	0.003	0.004	1.11	0.26
**Second year**					
Age	24	–0.008	0.01	0.57	0.56
Relationship length	18	0.001	0.002	0.54	0.58

*K, number of included samples.*

#### Relationship Length

We ran a similar analysis for relationship length (see Table 3). The effect of relationship length on the decrease in marital satisfaction was also non-significant (*b* = 0.003, S.E. = 0.003, *p* = 0.26).

### Marital Satisfaction in the Second Year Postpartum in Parents’ Samples (T1 to T2)

For testing the third hypothesis, we calculated whether the parents’ satisfaction decreases between the first postpartum assessment and a latter postpartum assessment (between 12 and 24 months postpartum, see Table 1 for details). We found no sufficient data to compute similar analyses in the non-parents’ sample.

Overall, the satisfaction decreased from T1 to T2, the results being significant (for mothers: Hedge’s g = –0.16, *p* < 0.001; for fathers: Hedge’s g = –0.14, *p* < 0.05). The effect sizes were small. The samples were heterogeneous (for mothers: *Q* = 158.47, *p* < 0.001, *I*^2^ = 91.17%; for fathers: *Q* = 46.19, *p* < 0.001, *I*^2^ = 78.35%). The meta-regression results show that the time difference between T1 and T2 did not influence the decrease in marital satisfaction for men or for women.

Moderator analyses were also conducted for the second year postpartum.

#### Gender

The results for mothers and fathers are displayed in Table 1. However, the differences between them were not significant (*Q* = 0.12, *p* = 0.72) (see Table 2).

#### Measurement

The decrease was weaker in the studies that used DAS compared to the studies that used other instruments to assess marital satisfaction (*Q* = 6.50, *p* = 0.01) (see Table 2).

#### Age

Age did not moderate the decrease in marital satisfaction from T1 to T2 (*b* = –0.008, S.E. = 0.01, *p* = 0.56) (see Table 3).

#### Relationship Length

The length of the relationship did not moderate the decrease in marital satisfaction from T1 to T2 (*b* = 0.001, S.E. = 0.002, *p* = 0.58) (see Table 3).

### Marital Satisfaction Reported by Parents in the First 2 Years Postpartum (T0 to T2)

Additionally, we calculated the effect sizes for the overall satisfaction decrease between prepartum and 24 months postpartum. The satisfaction decreased from T0 to T2, the result being significant (Hedge’s g = –0.37, *p* < 0.001). The effect size was medium (see Table 1 for more information). The sample was heterogeneous (*Q* = 367.29, *p* < 0.001, *I*^2^ = 93.19%).

### Cross-Partner Associations

We tested the forth hypothesis regarding the interdependence effect in the samples that came from the studies that used dyadic samples for the first year postpartum. For this, we computed a meta-regression where the effect sizes for decrease in one partner’s satisfaction were entered as a predictor for the decrease in the other partner’s satisfaction. The results were significant for both men (*b* = –0.68, S.E. = 0.08, *p* < 0.001) and women (*b* = 0.87, S.E. = 0.10, *p* < 0.001). The satisfaction of one partner has a steeper decline when the satisfaction of the other partner also has a more pronounced decrease.

### Publication Bias

To verify the publication bias, we applied Egger’s *T*-test. We obtained a value of –3.85 (*p* < 0.001), which, at first sight, would indicate a significant publication bias. However, using the trim and fill method, we found that by adding four studies, the adjusted value indicates a Hedge’s g score of –0.28, which does not differ from the initial result. In addition, after applying the Orwin Fail-safe N method, we noticed that another 367 studies would be needed to indicate a difference of –0.05 for the meta-analytical result to become zero. Thus, we found no significant publication bias.

## Discussion

We conducted a meta-analysis on longitudinal studies that investigated marital satisfaction during the transition to parenthood. The 97 samples of parents and 9 samples of non-parents from 49 empirical studies that fit our criteria allowed us to test the proposed hypotheses and achieve the meta-analysis’s aims. Our first aim was to deepen the trajectory of marital satisfaction in the first and in the second year postpartum for mothers and fathers, and to compare the parents’ trajectory with the non-parents’ trajectory of marital satisfaction for a similar period of marriage. The second goal is to examine the cross-partner associations of decline in marital satisfaction, and the third is to investigate the potential moderators of a decrease in postpartum marital satisfaction.

### Marital Satisfaction in the First Year Postpartum

Firstly, we verified whether marital satisfaction suffers a decrease from pregnancy up to 12 months postpartum for both men and women (H1). The results of the present meta-analysis indicate a medium decline in marital satisfaction for mothers and fathers in this period, thus our first hypothesis was confirmed. Then, we investigated the decrease of marital satisfaction in non-parents having a similar length of their relationship. For non-parents, the results reveal a small deterioration in marital satisfaction. Thus, our third hypothesis was confirmed: the trajectories of marital satisfaction are different in parents and non-parents. The decrease is higher for parents compared to non-parents (H3). These present results differ from the Mitnick et al. (2009) meta-analysis, which illustrated a small decrease in marital satisfaction for first-time parents and non-parents from pregnancy to the first 11 months after birth, respectively, for a similar duration of their relationship.

A drop in marital satisfaction during the first year of parenthood has been widely reported in the literature (Doss et al., 2009; Simonelli et al., 2016; Bäckström et al., 2018). This decrease may prove the difficulties that first-time parents go through in the transition to parenthood (Bäckström et al., 2018). A plethora of factors could determine deterioration in marital satisfaction, such as the change from a system of spouses without children to a system of parents with a child (Minuchin, 1974; Simonelli et al., 2016), the stress generated by childcare (Condon et al., 2004), reduced postpartum communication and responsiveness (Perren et al., 2005) as well as multiple activities performed simultaneously (Baxter et al., 2008). Once the partners become parents, they experience more marital conflicts and more dissatisfaction toward the marriage stage (Perren et al., 2005; Bouchard et al., 2006). Our results seem to support the idea that marital satisfaction’s decline is significant and quite abrupt for up to 1 year postpartum (Lawrence et al., 2008).

Concerning the newlyweds without children, our result reflects a middle point between the previous theories. The small decrease in marital satisfaction, identified by our meta-analysis, is interposed between “gradual disillusionment,” “the honeymoon is over,” or the “honeymoon followed by blandness” models (Kurdek, 1998; Huston et al., 2001; Aron et al., 2002), and the “enduring dynamics model” (Huston et al., 2001). The first ones state that the husbands begin their matrimonies with a high level of satisfaction, the relationship satisfaction decreasing over time. The last model supports the idea of marital satisfaction stability over time. Moreover, recent research shows that, in general, couples report a relatively constant level of marital satisfaction during the first years. Exceptions are those who start their marriage with a low level of marital satisfaction, but their number is considerably lower than those who report medium and high satisfaction levels (Birditt et al., 2012; Proulx et al., 2017; Williamson and Lavner, 2020). Future investigation in newlyweds should more often include samples of parents and non-parents in longitudinal designs in order to draw more reliable causal conclusions.

### Marital Satisfaction in the Second Year Postpartum

From the first to the second year postpartum, marital satisfaction has a small decrease for first-time parents, with a similar decline for mothers and fathers, confirming the second hypothesis, that the decrease in marital satisfaction continues between 12 and 24 months postpartum for both men and women (H2). Unfortunately, the studies included in this meta-analysis did not present enough data to allow us to measure the course of marital satisfaction in couples without children for a similar interval of time.

Previous research supports our findings regarding the decline in marital satisfaction for the second year postpartum (Figueiredo and Conde, 2015). The difficulties that partners face during the transition to parenthood does not stop after the first year postpartum. Some aspects of the transition to parenthood, like the increase in marital conflict (Christopher et al., 2015), negative communication and problem intensity (Doss et al., 2009; Figueiredo and Conde, 2015), parental stress (Eller et al., 2019; Gou et al., 2019) and couple intimacy (Figueiredo et al., 2018) influence the trajectory of marital satisfaction from first to the second year postpartum.

### Marital Satisfaction in the First 2 Years Postpartum Globally

Additionally, our results show that marital satisfaction has a medium decrease from pregnancy to 24 months postpartum. These data converge with those obtained by Twenge et al. (2003) and with several longitudinal studies using multiple waves (e.g., Doss et al., 2009; Hirschberger et al., 2009; Simonelli et al., 2016). First-time parents can show a substantial increase in hostility, disagreement and problem severity several years after the infant’s birth. The magnitude of negative post-birth relationship changes is unexpected for parents; the most likely reason is the exacerbation of stressors that are not well controlled during the transition to parenthood (Doss et al., 2009) which affects marital satisfaction (Lu, 2006). Referring to directions of change proposed by Lawrence et al. (2008), our results support the first one, consisting in a significant and qualitative decrease in marital satisfaction to the detriment of the temporary decline. According to our meta-analysis, the decrease in marital satisfaction is significant, of medium intensity, and characterizes at least the first 2 years after the birth of the first child. Attachment theory has shown that in difficult times anxiety and avoidance tend to increase, especially affecting couples in which one or both partners are more insecurely attached (Feeney et al., 2003). Higher insecurity is associated with lower affect regulation, interpersonal functioning, and relationship satisfaction (Mikulincer and Shaver, 2007). Furthermore, several studies have investigated the course of marital satisfaction many years after the first baby arrives and concluded that the decline of marital satisfaction remains at 4, 8, and 15 years after becoming a parent (Doss et al., 2009; Simonelli et al., 2016).

### Cross-Partner Associations

In order to test our fourth hypothesis, data showed that a steeper decrease in one partner’s satisfaction is associated with the steeper decrease in the other partner’s level of satisfaction (H4). Thus, there are significant cross-partner association effects between prepartum and postpartum marital satisfaction. Our results represent an extension of previous studies indicating the interdependence effect in couples, that found that couples report similar levels of marital satisfaction during the transition to parenthood (Elek et al., 2003; Don and Mickelson, 2014), and one partner’s marital satisfaction predicted the other partner’s relationship quality at a later time (Lee, 2017). It seems that the two partners go through the same marital satisfaction patterns in the first year postpartum (Figueiredo and Conde, 2015) and see their couple relationship in related ways (Elek et al., 2003). This mutual influence can be explained by the couple dynamic. Both parents also experience several changes once the first child is born and becomes more easily influenced by their partner. Birth, confinement, breastfeeding, job suspension, diminishing independence and reduced personal freedom make women more sensitive to their partner’s characteristics. A supportive partner’s approach helps them get over these changes more easily (Rauch-Anderegg et al., 2020), while a stressed, unsupportive, depressed or an anxious spouse impacts the individual’s marital satisfaction (Don and Mickelson, 2014) and makes the transition more difficult. Although some data shows that the mother’s behavior had no impact on the father’s marital satisfaction (Rauch-Anderegg et al., 2020), it seems that some latent variables of the mothers have a substantial impact. For example, a high level of a mother’s daily stress and low self-esteem are related to a decline in the father’s marital satisfaction during the transition to parenthood (Meijer and van den Wittenboer, 2007; Don and Mickelson, 2014).

### Moderators of Decrease in Marital Satisfaction

Although some evidence suggests possible moderators of decrease in marital satisfaction in first-time parents, like gender (Don and Mickelson, 2014), age (Lederman et al., 1981; Zare et al., 2014), and length of relationship (O’Brien and Peyton, 2002; Doss et al., 2009; Lavner et al., 2020), our results reveal that only the type of instrument used presents a significant moderation of decline of marital satisfaction in the second postpartum year. So, our last hypothesis, that the decline in marital satisfaction is moderated by gender, age, relationship length, and the instrument used for measuring marital satisfaction (H5), was partially confirmed. The decline was smaller in studies that used the Dyadic Adjustment Scale (DAS, Spanier, 1976) compared to the studies that used other instruments to assess marital satisfaction. The scales measuring marital satisfaction include a conglomerate of items comprising evaluative judgments about marital quality, reports of specific behaviors and general interaction patterns (Bradbury et al., 2000), the number, scoring and relevance of the items varying greatly from one instrument to another (Fincham and Beach, 2006). Being one of the first scales built to assess marital satisfaction, it is possible that DAS, which does not include all the negative aspects affecting first-time parents, increases contamination to social desirability / acquiescence response bias in less difficult times compared to other scales. Moreover, this discrepancy concerning the moderating role of the couple satisfaction measure may indicate that after the first year postpartum, the parents are more sensitive to some aspects of their marital relationship, like love, sensuality or sexuality. These aspects of marital satisfaction are included in several marital satisfaction scales (e.g., Quality of Dyadic Relationship; Ahlborg et al., 2009; Relationship Assessment Scale; Hendrick, 1988) but not in DAS.

Concerning gender as a possible moderator, our results show a similar trajectory for men and women across all time points, thus contradicting the idea that the transition to parenthood impacts women’s marital satisfaction more than men’s (Twenge et al., 2003). Our results are in line with other research’s results indicating that parents do not differ in their trajectory of postpartum marital satisfaction, inferring that husbands have similar perspectives when it comes to their relationship (Elek et al., 2003; Don and Mickelson, 2014).

Therefore, our meta-analysis confirms the previous line of findings that sustain the marital satisfaction decline once the first child is born. Previously, little was known about the trajectory of marital satisfaction from 12 to 24 months postpartum. Our work covers this gap and reveals a low decrease in first-time parents’ marital satisfaction. Also, for the first time, our results offer a global summary of cross-partner associations in the trajectory of postpartum marital satisfaction. Concerning the therapeutical inferences, we want to conclude with three practical implications. First, when delivering parenthood educational training to couples, practitioners should know that pre-pregnancy levels of marital satisfaction will tend to contract spouses’ evaluations of the marriage after the baby is born (Lawrence et al., 2008), but the decline is lower when parents have realistic expectations about parenthood (Menéndez et al., 2011). Second, professionals should be aware that a medium decrease in marital satisfaction is common in first-time parents. Knowing that transition to parenthood moderately affects marital satisfaction for the average couple and that the couple’s satisfaction before the birth of the first child is a strong predictor of how couples manage this transition suggests the need to build dyadic interventions before the baby arrives and a long time after. Third, family counselors should know that marital satisfaction continues to decrease in the second year after childbirth. A realistic view of marital quality during the transition to parenthood should help souses manage this period better.

### Limitations and Furture Directions

Several limitations should be considered when interpreting the findings of the present study. The first limitation concern with the number of the included non-parent samples. Also, the some parents’ studies reported a common mean of marital satisfaction for married and unmarried participants. Future research should use independent means for the two categories of participants, considering that couples who have a child after marriage report higher marital satisfaction than couples who become parents before marriage (Lavner et al., 2020). Secondly, not all included studies show averages of marital satisfaction at exactly 12 or 24 months postpartum, which is why we chose the averages reported to the nearest proposed time. Also, even if our study does not suffer from publication bias, it does not contain unpublished data. A prospective investigation should introduce unpublished data, which will lead to an increasing number of studies.

## Conclusion

Summarizing our results, the drop in marital satisfaction during the first year of parenthood is medium for both mothers and fathers. In the same period of their marriage, non-parents reported only a small decline. The satisfaction’s decline continues in mothers and fathers in the second year postpartum to a lesser extent. Also, the cross-partner associations of marital satisfaction decrease were noticed during the first postartum year. Although we found no publication bias, no moderation effect of the participant’s age, gender or of the length of their relationships, the instrument of measurement moderates the marital satisfaction decrease in the second year postpartum.

## Data Availability Statement

The datasets presented in this study can be found in online repositories. The names of the repository/repositories and accession number(s) can be found below: https://osf.io/xp3hk/files/.

## Ethics Statement

Ethical review and approval was not required for the study on human participants in accordance with the local legislation and institutional requirements. Written informed consent for participation was not required for this study in accordance with the national legislation and the institutional requirements.

## Author Contributions

IB and MT contributed to all phases of the article. OC contributed to the design phase of the manuscript, and collection and analysis of the data. All authors contributed to the article and approved the submitted version.

## Conflict of Interest

The authors declare that the research was conducted in the absence of any commercial or financial relationships that could be construed as a potential conflict of interest.

## Publisher’s Note

All claims expressed in this article are solely those of the authors and do not necessarily represent those of their affiliated organizations, or those of the publisher, the editors and the reviewers. Any product that may be evaluated in this article, or claim that may be made by its manufacturer, is not guaranteed or endorsed by the publisher.

## Footnotes

1
https://osf.io/j3v9z
