# Supplementary material for: Transition to Parenthood and Marital Satisfaction: A Meta-Analysis
Source: Front Psychol. 2022 Jul 20;13:901362. doi: 10.3389/fpsyg.2022.901362 (PMC9350520; doi:10.3389/fpsyg.2022.901362)
Supplement: Supplementary file 1 [file Data_Sheet_1.pdf]

Studies included in meta-analysis

Ahmad, G., & Najam, N. (1998). A study of marital adjustment during first transition to parenthood. *Journal of Behavioural Sciences, 9(1-2)*, 67–86.

Bäckström, C., Kåreholt, I., Thorstensson, S., Golsäter, M., & Mårtensson, L. B. (2018). Quality of couple relationship among first-time mothers and partners, during pregnancy and the first six months of parenthood. *Sexual & Reproductive Healthcare, 17*, 56–64. https://doi.org/10.1016/j.srhc.2018.07.001

Biehle, S. N., & Mickelson, K. D. (2012). First-time parents' expectations about the division of childcare and play. *Journal of Family Psychology, 26*(1), 36–45. https://doi.org/10.1037/a0026608

Bouchard, G., Boudreau, J., & Hébert, R. (2006). Transition to Parenthood and Conjugal Life: Comparisons Between Planned and Unplanned Pregnancies. *Journal of Family Issues, 27*(11), 1512–1531. https://doi.org/10.1177/0192513X06290855

Bower, D., Jia, R., Schoppe-Sullivan, S. J., Mangelsdorf, S. C., & Brown, G. L. (2013). Trajectories of couple relationship satisfaction in families with infants: The roles of parent gender, personality, and depression in first-time and experienced parents. *Journal of Social and Personal Relationships*, *30*(4), 389–409. https://doi.org/10.1177/0265407512457656

Buist, A., Morse, C. A., & Durkin, S. (2003). Men's Adjustment to Fatherhood: Implications for Obstetric Health Care. *Journal of Obstetric, Gynecologic & Neonatal Nursing, 32*(2), 172–180. https://doi.org/10.1177/0884217503252127

Cairo, S., Darwiche, J., Tissot, H., Favez, N., Germond, M., Guex, P., de Roten, Y., Frascarolo, F., & Despland, J.-N. (2012). Family interactions in IVF families: Change over the transition to parenthood. *Journal of Reproductive and Infant Psychology, 30*(1), 5–20. https://doi.org/10.1080/02646838.2012.669830

Castellano, R., Velotti, P., Crowell, J. A., & Zavattini, G. C. (2014). The Role of Parents' Attachment Configurations at Childbirth on Marital Satisfaction and Conflict Strategies. *Journal of Child and Family Studies*, *23*(6), 1011–1026. https://doi.org/10.1007/s10826-013-9757-7

Christopher, C., Umemura, T., Mann, T., Jacobvitz, D., & Hazen, N. (2015). Marital Quality over the Transition to Parenthood as a Predictor of Coparenting. *Journal of Child and Family Studies*, *24*(12), 3636–3651. https://doi.org/10.1007/s10826-015-0172-0

Condon, J. T., Boyce, P., & Corkindale, C. J. (2004). The First-Time Fathers Study: A prospective study of the mental health and wellbeing of men during the transition to parenthood. *Australian and New Zealand Journal of Psychiatry*, *38*(1–2), 56–64. https://doi.org/10.1111/j.1440-1614.2004.01298.x

Coop Gordon, K., Roberson, P. N. E., Hughes, J. A., Khaddouma, A. M., Swamy, G. K., Noonan, D., Gonzalez, A. M., Fish, L., & Pollak, K. I. (2018). The Effects of a Couples-Based Health Behavior Intervention During Pregnancy on Latino Couples' Dyadic Satisfaction Postpartum. *Family Process*, *57*(3), 629–648. https://doi.org/10.1111/famp.12354

Cox, M. J., Paley, B., Burchinal, M., & Payne, C. C. (1999). Marital Perceptions and Interactions Across the Transition to Parenthood. *Journal of Marriage and the Family*, *61*(3), 611. https://doi.org/10.2307/353564

Don, B. P., & Mickelson, K. D. (2014). Relationship Satisfaction Trajectories Across the Transition to Parenthood Among Low-Risk Parents: Relationship Satisfaction Trajectories. *Journal of Marriage and Family*, *76*(3), 677–692. https://doi.org/10.1111/jomf.12111

Doss, B. D., Rhoades, G. K., Stanley, S. M., & Markman, H. J. (2009). The effect of the transition to parenthood on relationship quality: An 8-year prospective study. *Journal*

of Personality and Social Psychology, *96*(3), 601–619. https://doi.org/10.1037/a0013969

Dulude, D., Bélanger, C., Wright, J., & Sabourin, S. (2002). High-risk pregnancies, psychological distress, and dyadic adjustment. *Journal of Reproductive and Infant Psychology*, *20*(2), 101–123. https://doi.org/10.1080/02646830220134612

Dyrdal, G. M., Røysamb, E., Nes, R. B., & Vittersø, J. (2011). Can a Happy Relationship Predict a Happy Life? A Population-Based Study of Maternal Well-Being During the Life Transition of Pregnancy, Infancy, and Toddlerhood. *Journal of Happiness Studies*, *12*(6), 947–962. https://doi.org/10.1007/s10902-010-9238-2

Feldman, S. S., & Nash, S. C. (1984). The transition from expectancy to parenthood: Impact of the firstborn child on men and women. *Sex Roles*, *11*(1–2), 61–78. https://doi.org/10.1007/BF00287441

Figueiredo, B., Canário, C., Tendais, I., Pinto, T. M., Kenny, D. A., & Field, T. (2018). Couples' relationship affects mothers' and fathers' anxiety and depression trajectories over the transition to parenthood. *Journal of Affective Disorders*, *238*, 204–212. https://doi.org/10.1016/j.jad.2018.05.064

Fillo, J., Simpson, J. A., Rholes, W. S., & Kohn, J. L. (2015). Dads doing diapers: Individual and relational outcomes associated with the division of childcare across the transition to parenthood. *Journal of Personality and Social Psychology*, *108*(2), 298–316. https://doi.org/10.1037/a0038572

Gameiro, S., Nazaré, B., Fonseca, A., Moura-Ramos, M., Canavarro, M. C. (2011). Changes in marital congruence and quality of life across the transition to parenthood in couples who conceived spontaneously or with assisted reproductive technologies.*Fertility and Sterility*, *96*(6), 1457–1462. https://doi.org/10.1016/j.fertnstert.2011.09.003

Gjerdingen, D. K., & Center, B. (2002). A Randomized Controlled Trial Testing the Impact of a Support/Work-Planning Intervention on First-Time Parents' Health, Partner Relationship, and Work Responsibilities. *Behavioral Medicine*, *28*(3), 84–91. https://doi.org/10.1080/08964280209596045

Gou, L. H., Duerksen, K. N., & Woodin, E. M. (2019). Coercive control during the transition to parenthood: An overlooked factor in intimate partner violence and family wellbeing? *Aggressive Behavior*, *45*(2), 139–150. https://doi.org/10.1002/ab.21803

Grote, N. K., & Clark, M. S. (2001). Perceiving unfairness in the family: Cause or consequence of marital distress? *Journal of Personality and Social Psychology*, *80*(2), 281–293. https://doi.org/10.1037/0022-3514.80.2.281

Harwood, K., McLean, N., & Durkin, K. (2007). First-time mothers' expectations of parenthood: What happens when optimistic expectations are not matched by later experiences? *Developmental Psychology, 43*(1), 1–12. https://doi.org/10.1037/0012-1649.43.1.1

Hirschberger, G., Srivastava, S., Marsh, P., Cowan, C. P., & Cowan, P. A. (2009). Attachment, marital satisfaction, and divorce during the first fifteen years of parenthood. *Personal Relationships*, *16*(3), 401–420. https://doi.org/10.1111/j.1475-6811.2009.01230.x

Hock, E., Schirtzinger, M. B., Lutz, M. B., & Widaman, K. (1995). Maternal depressive symptomatology over the transition to parenthood: Assessing the influence of marital satisfaction and marital sex role traditionalism. *Journal of Family Psychology*, *9*(1), 79–88. https://doi.org/10.1037/0893-3200.9.1.79

Kestler-Peleg, M., Lavenda, O., Keren-Leneman, S., & Ginzburg, K. (2019). Spousal Relationship (E) Quality and Adjustment to Motherhood in Israel. *Journal of Family Issues*, *40*(11), 1561–1578. https://doi.org/10.1177/0192513X19842590

Kurdek, L. A. (1998). The nature and predictors of the trajectory of change in marital quality over the first 4 years of marriage for first-married husbands and wives. *Journal of Family Psychology*, *12*(4), 494–510. https://doi.org/10.1037/0893-3200.12.4.494

Lawrence, E., Rothman, A. D., Cobb, R. J., Rothman, M. T., & Bradbury, T. N. (2008). Marital satisfaction across the transition to parenthood. *Journal of Family Psychology*, *22*(1), 41–50. https://doi.org/10.1037/0893-3200.22.1.41

Levy-Shiff, R. (1994). Individual and contextual correlates of marital change across the transition to parenthood. *Developmental Psychology*, *30*(4), 591–601. https://doi.org/10.1037/0012-1649.30.4.591

Lu, L. (2006). The transition to parenthood: Stress, resources, and gender differences in a Chinese society. *Journal of Community Psychology*, *34*(4), 471–488. https://doi.org/10.1002/jcop.20110

Markman, H. J., & Kadushin, F. S. (1986). Preventive effects of Lamaze training for first-time parents: A short-term longitudinal study. *Journal of Consulting and Clinical Psychology*, *54*(6), 872–874. https://doi.org/10.1037/0022-006X.54.6.872

Meijer, A. M., & van den Wittenboer, G. L. H. (2007). Contribution of infants' sleep and crying to marital relationship of first-time parent couples in the 1st year after childbirth. *Journal of Family Psychology*, *21*(1), 49–57. https://doi.org/10.1037/0893-3200.21.1.49

Mellingen, S., Torsheim, T., & Thuen, F. (2015). Effect of Prepregnancy Alcohol Consumption on Postpartum Relationship Satisfaction and Divorce among Norwegian Mothers. *Substance Abuse: Research and Treatment*, *9s2*, SART.S23543. https://doi.org/10.4137/SART.S23543

Menéndez, S., Hidalgo, M. V., Jiménez, L., & Moreno, M. C. (2011). Father Involvement and Marital Relationship during Transition to Parenthood: Differences between Dual and

Single-Earner Families. *The Spanish Journal of Psychology*, *14*(2), 639–647. https://doi.org/10.5209/rev_SJOP.2011.v14.n2.12

Mortensen, Ø., Torsheim, T., Melkevik, O., & Thuen, F. (2012). Adding a Baby to the Equation. Married and Cohabiting Women's Relationship Satisfaction in the Transition to Parenthood. *Family Process*, *51*(1), 122–139. https://doi.org/10.1111/j.1545-5300.2012.01384.x

Pancer, S. M., Pratt, M., Hunsberger, B., & Gallant, M. (2000). Thinking Ahead: Complexity of Expectations and the Transition to Parenthood. *Journal of Personality*, *68*(2), 253–279. https://doi.org/10.1111/1467-6494.00097

Porter, C. L., & Hsu, H.-C. (2003). First-time mothers' perceptions of efficacy during the transition to motherhood: Links to infant temperament. *Journal of Family Psychology*, *17*(1), 54–64. https://doi.org/10.1037/0893-3200.17.1.54

Rauch-Anderegg, V., Kuhn, R., Milek, A., Halford, W. K., & Bodenmann, G. (2020). Relationship Behaviors across the Transition to Parenthood. *Journal of Family Issues*, *41*(4), 483–506. https://doi.org/10.1177/0192513X19878864

Reid, C. A., Worthington, E. L., Garthe, R. C., Davis, D. E., Hook, J. N., Van Tongeren, D. R., & Griffin, B. J. (2018). Actor–partner interdependence of humility and relationship quality among couples transitioning to parenthood. *The Journal of Positive Psychology*, *13*(2), 122–132. https://doi.org/10.1080/17439760.2016.1233349

Rholes, W. S., Eller, J., Simpson, J. A., & Arriaga, X. B. (2021). Support Processes Predict Declines in Attachment Avoidance Across the Transition to Parenthood. *Personality and Social Psychology Bulletin*, *47*(5), 810–825. https://doi.org/10.1177/0146167220948706

Rholes, W. S., Simpson, J. A., Campbell, L., & Grich, J. (2001). Adult attachment and the transition to parenthood. *Journal of Personality and Social Psychology*, *81*(3), 421–435. https://doi.org/10.1037/0022-3514.81.3.421

Schulz, M. S., Cowan, C. P., & Cowan, P. A. (2006). Promoting healthy beginnings: A randomized controlled trial of a preventive intervention to preserve marital quality during the transition to parenthood. *Journal of Consulting and Clinical Psychology*, *74*(1), 20–31. https://doi.org/10.1037/0022-006X.74.1.20

Simonelli, A., Parolin, M., Sacchi, C., De Palo, F., & Vieno, A. (2016). The Role of Father Involvement and Marital Satisfaction in the Development of Family Interactive Abilities: A Multilevel Approach. *Frontiers in Psychology*, *7*. https://doi.org/10.3389/fpsyg.2016.01725

Terry, D. J., McHugh, T. A., & Noller, P. (1991). Role dissatisfaction and the decline in marital quality across the transition to parenthood. *Australian Journal of Psychology*, *43*(3), 129–132. https://doi.org/10.1080/00049539108260136

Trillingsgaard, T., Baucom, K. J. W., & Heyman, R. E. (2014). Predictors of Change in Relationship Satisfaction during the Transition to Parenthood: Predictors of Change in Relationship Satisfaction. *Family Relations*, *63*(5), 667–679. https://doi.org/10.1111/fare.12089

Tucker, P., & Aron, A. (1993). Passionate Love and Marital Satisfaction at Key Transition Points in the Family Life Cycle. *Journal of Social and Clinical Psychology*, *12*(2), 135–147. https://doi.org/10.1521/jscp.1993.12.2.135

van Egeren, L. A. (2004). The development of the coparenting relationship over the transition to parenthood. *Infant Mental Health Journal*, *25*(5), 453–477. https://doi.org/10.1002/imhj.20019

Wright, P. J., Henggeler, S. W., & Craig, L. (1986). Problems in paradise?: A longitudinal examination of the transition to parenthood. *Journal of Applied Developmental Psychology*, *7*(3), 277–291. https://doi.org/10.1016/0193-3973(86)90035-3
